# Afrocentrism, national interest and citizen welfare in Nigeria’s foreign policy maneuvers

**DOI:** 10.12688/f1000research.25036.1

**Published:** 2020-08-18

**Authors:** George Chimdi Mbara, Nirmala Gopal

**Affiliations:** 1 School of Applied Human Sciences, University of Kwa-Zulu Natal, Durban, KwaZulu-Natal, 4041, South Africa

**Keywords:** Afrocentrism, Citizen welfare, Foreign policy, National interest

## Abstract

**Background:** Nigeria’s former Prime Minister, Sir Abubakar Tafawa Balewa, in his addresses of August and October 1, 1960, declared Africa as the centrepiece of Nigeria’s foreign policy. This policy thrust has remained a constant variable in the country’s diplomatic engagements over the years. The doctrine of Afrocentrism is predicated on the supposed manifest leadership role placed on Nigeria by nature. This made her leaders define Africa’s interest as Nigeria’s national interest, a development that has been contended to have no empirical bearing on the welfare of Nigerians thereby generating intense scrutiny. Consequently, this study evaluates the impact of Nigeria’s Afrocentric foreign policy thrust on the welfare of the ordinary Nigerians. The study further analyses the country’s gravitation towards citizen-centred diplomacy in 2007. These will help in comprehending the interaction between national interest and foreign policy in Nigeria, and to identify whose interests have been protected the most in Nigeria’s foreign policy pursuit – that of the ordinary citizens or the elites?

**Methods:** Through the qualitative research method, in-depth interviews (IDIs) were conducted with Key Informants (KIs) for data collection. Responses from field study are merged with other primary and secondary sources of data to provide an incisive and balanced analysis that is premised on political realism.

**Results:** Findings indicate that Nigeria’s international generosity and leadership role has never been predicated on a clear vision of national interest. Notwithstanding the flaws in Nigeria’s foreign policy over the years, this study also discovered that the outcome has not been a total failure as some respondents maintain.

**Conclusions:** With the nation’s gravitation towards citizen-centred diplomacy, it is hoped that the country will put the interest of its citizens first in her policy pursuits.

## Introduction

Following the addresses by Nigeria’s former Prime Minister, Sir Abubakar Tafawa Balewa in August and on October 1, 1960, which pronounced Africa as the centrepiece of Nigeria’s foreign policy, this policy thrust has remained a constant variable in the country’s diplomatic engagements over the years ( Adeniji, 2005; Akinterinwa, 2004; Dan-Fulani, 2014; Folarin, 2013; Jega, 2010; Saliu, 2006). Commenting on Afrocentrism, King (1996) cited in Folarin (2013), describes Africa-centred diplomacy as a political construct in which a country perceives the interests and welfare of the African region as critical to its interests and concerns as a nation. He describes it as an existential principle that sees a nation-state display a generous and magnanimous disposition towards African nations in need. Corroborating this view, Mazrui (2006) sees Nigeria’s Afrocentric policy as a Pan-Africanist worldview that has underscored its foreign policy since independence. By this disposition, Nigeria’s foreign policy gravitated around Africa and issues affecting the region received full attention ahead of matters outside the continent as exemplified in the formation of OAU and in solving the 1960–1965 Congolese crisis.

In her ‘rescue operations’, it is estimated that Nigeria has spent over 60 billion US dollars in financial assistance to countries in Africa and the Caribbean, not to mention the human cost ( Fawole, 2003). No exact estimate has been made on the human cost of these operations. Its Africa-centred policy has been pursued at a very huge cost to the country and its people since independence. As early as 1960, Nigeria became actively involved in achieving international peace and security by contributing to global and regional peacekeeping, making her one of the highest contributors to United Nations peace operations. Among other operations, Nigeria supplied troops to DR Congo, Liberia, Sierra Leone, Somalia, Sudan, Sao Tome and Cote d’Ivoire.

The doctrine of Afrocentrism is predicated on the supposed manifest leadership role placed on Nigeria by nature, which made her leaders define Africa’s interest as Nigeria’s national interest ( Warner, 2017); a development that has been contended to have no empirical bearing on the welfare of Nigerians thereby generating intense scrutiny ( Dan-Fulani, 2014; Folarin, 2010; Mbara, 2019). In its Africa-first policy, did Nigeria play the felt leadership role and did other African states recognize and acknowledge the claim? Suffice it to say that it is one thing to see yourself as a leader, and it another thing to be accepted as one by others. Through the various military and civilian administrations since independence, this has remained a guiding principle of the nation’s policy constructs, applied at various degrees. To make matters worse, even with the country’s generosity, it is certain that her economic and social sacrifice has not yielded commensurate investments in human resource and capital development at home. Likewise, Nigeria’s “Big Brother” status has hardly been acknowledged, a clear indication that her claim to hegemony in Africa may have faded away. Besides that, the country’s respect and foreign image has been deteriorating as her citizens are constantly molested, harassed, unjustly detained and even killed abroad ( Dan-Fulani, 2014; Fawole, 2003; Warner, 2017).

Consequently, this study evaluates the impact of Nigeria’s Afrocentric foreign policy thrust on the welfare of the ordinary Nigerians. The study further analyses the country’s gravitation towards a citizen-centred diplomacy in 2007. These will help in comprehending the interaction between national interest and foreign policy in Nigeria, and to identify whose interests have been protected the most in Nigeria’s foreign policy pursuit – that of the ordinary citizens or the elites? Responses from field study are merged with other primary and secondary sources of data to provide an incisive and balanced analysis.

## Methods

The qualitative research method was used in this study. This research method involved in-depth Interviews (IDIs) with key informants (KIs) which were conducted for data collection. Through purposive sampling, the research population included relevant stakeholders in the country’s foreign policy formulation and implementation organs. Semi-structured in-depth interviews with “strategic informants” guided the data collection for the study as it relates to the various themes under investigation. Keen attention was paid to the responses of the participants to identify new areas of inquiry that are directly connected to the phenomenon under investigation.

This study took place in August and September of 2017 as a doctoral research thesis on “Nigeria’s Quest for a Permanent Seat on an Expanded UN Security Council: What Relevance for Domestic Factors?” The sample population was categorized into two sets of participants: the first includes a spokesperson from the Ministry of Foreign Affairs, and six senior officials/resource persons from the National Institute for Policy and Strategic Studies (NIPSS), Kuru, Plateau State, Nigeria. This first category of respondents is identified as “Group A.” The second category of respondents, identified as “Group B,” includes two academics and two postgraduate students from University of Jos who spoke on behalf of Nigerian students; one respondent from of the Federal Ministry of Information and Culture, and four “ordinary Nigerians” were carefully chosen from the petty traders, artisans and unemployed persons in the country to make up the sample population. These “ordinary Nigerians” represent the *vox populi*, they helped the researcher feel the pulse of the people on the street about the investigation. They were chosen through purposive sampling from recommendations of knowledgeable people and out of convenience for the researcher. The “ordinary Nigerians” were approached by the investigator in person. This brings the interviews conducted to a combined total of 16. This sample size was chosen in view of the principle of data saturation, that is, to avoid unnecessary repetition of data. These constitute the sample population for the primary source of data for the study. Each respondent was met at their respective workplaces for the interviews and each session lasted for about 25 to 35 minutes, depending on the flow of information. Interviews were recorded by the researcher using a smartphone and notes were taken during the sessions. The interview guides for each interview are available as *Extended data* ( Mbara, 2020).

The data gathered were evaluated using content analysis, textual criticism and descriptive-historical analysis. The analyses were situated within the purview of the various research questions and in the light of the realist perspective on international politics. The study also utilised secondary sources of data archival materials, ranging from Nigerian government official documents, academic journals, newspapers, textbooks, conference papers, as well as reliable and verifiable internet materials.

## Ethical issues

Ethical approval for this study was granted by the Humanities & Social Sciences Ethics Committee, University of KwaZulu-Natal, South Africa with protocol reference number: HSS/1033/017D. Prior to the collection of data for this study, gatekeeper’s permits were secured from the National Institute for Policy and Strategic Studies, Kuru and the Federal Ministry of Foreign Affairs, Abuja. For other respondents in the second category, an informed consent was received from each of them to participate in the study. In the informed consent form, respondents were assured that their views will be presented anonymously in the study, neither their names nor personal details were going to be disclosed while presenting the data. For this reason, respondents are represented with pseudonyms in this report and access to the interview recordings are restricted since they identify each respondent’s personal details. However, to replicate the study, an application can be made to the Humanities & Social Sciences Ethics Committee of the University of KwaZulu-Natal for access to the data.

## Results

### Dialectics of Nigeria’s foreign aid, national interest and citizens welfare

The diplomacy of aid assistance in international relations has variously been explained ( Dan-Fulani, 2014; Holsti, 1994; Mailafia, 2010). The realist perspective provides a concise explanation for the games nations play, namely, that countries offer aid to others based on calculated self-interest. Interests of this nature may be medium-term, or long-term, explicit, covert, or obscure. From this viewpoint, aid is one of the arsenals of economic diplomacy being deployed in the quest for national interest. Similarly, aid is also perceived as a form of imperialism. At the summit of the Cold War, when aid was used as part of the arsenals of ‘informal empire’, scholars like Hayter (1985) cited in Mailafia (2010), propagated the idea of “aid as imperialism,” a mechanism for courting friends as well as cajoling allies who were at the margins of the world capitalist system. On the other hand, states can and do offer aid for altruistic intentions. Wealthy nations come to the assistance of poorer neighbours for the purpose of charity and generosity. Likewise, in moments of a humanitarian crisis, conflict or natural disasters, most of this aid comes from an altruistic intention.

Nigeria has always seen itself as the regional hegemon in Africa, to the extent that the quest for Pax-Nigeriana has been a motivating factor, to varying degrees, of every Nigerian administration since independence, especially the regimes of 1960–1993 ( Nuamah, 2003). To this end, Nigeria’s aspiration for continental leadership since independence is key to understanding some pivotal features of Nigeria’s foreign policy. Moreover, Pax-Nigeriana and Afrocentrism are structured around “concentric circles of foreign policy.”

*Four concentric circles underlie Nigeria’s grand strategy – from the innermost to the outermost. The innermost level deals with Nigeria’s relationship with its immediate neighbours (Sao Tome and Principe, Cameroon, Chad, Benin Republic, Niger and Equatorial Guinea). This is followed by Nigeria’s relations with other West African countries, Nigeria and the rest of Africa, then Nigeria in the world.* (Respondent from the Federal Ministry of Foreign Affairs, Abuja.)

Analysts believe that Nigeria’s Afrocentrism was largely responsible for its dogged fight in dismantling apartheid ( Adeniji, 2005; Amujiri *et al.*, 2015; Dan-Fulani, 2014; Osuntokun, 2005). Nigeria internationalized the issue and made a huge financial commitment to fight the social/political menace. The robust economy of the 1970s won Nigeria the respect and credibility as the most populous black nation on earth and a credible voice to speak for Africa. This no doubt made the world to accept Nigeria’s proposal to establish anti-apartheid committees in both the OAU (now AU) and the UN with the country having permanent chairs on the committees ( Gambari, 1997). Nigeria successfully got countries to boycott the 13th Commonwealth games and sanctioned countries and companies that continued to deal with the apartheid government ( Ade-Ibijola, 2015; Amujiri *et al*., 2015; Hamil & Spemce, 1994).

Nigeria played an active role in the liberation of Southern Africa and the eradication of apartheid and colonialism, as well as supporting needy African countries with financial, material and technical aid over the years ^1^ ( Ade-Ibijola, 2014; Adeniji, 2005; Akpotor & Agbebaku, 2010; Dan-Fulani, 2014; Fawole, 2000; Kyenge, 2015). Similarly, Osuntokun (2005) commenting on the role Nigeria played in African decolonization efforts stated “[Nigeria] … sacrificed the goodwill of the West and economic development in order to see to the total liberation of Africa.” In the same vein, Garba (1987:101) asserts:

Nigeria… made enemies of erstwhile friends – all on account of their attitude towards the South Africa question. We have formulated economic policies that have sometimes been detrimental to our development because of our commitment to the eradication of apartheid.

In addition, Garba noted that the country lost the enormous sum of $45 billion over a period of 15 years for its embargo on exporting oil to apartheid South Africa. These facts bring us back to one of the research questions, what is the interaction between national interest and foreign policy in Nigeria, and does the interest represent the aspirations of its citizens? Going by the realist theoretical framework, which this study is built on, the obvious answer is No!

Notwithstanding these successes, remarking on the Afrocentric policy, Amao & Uzodike (2015:10) averred:

Regardless of these successes, this Africa-centred foreign policy concentration has not been without flaws. These flaws were soon to become evident in the downturn experienced by the country in its hitherto strong and viable economy and in the neglect of its domestic responsibilities, specifically the fulfilment of the social obligations expected of a government to its people. The resultant effect of this has been a steady decline in the nation’s oil revenue owing to a culture of poor maintenance, corruption and the extensive projects executed by Nigeria in other African countries.

Nigeria’s international generosity has never been predicated on a clear vision of national interest ( Mailafia, 2010). The country’s interventionist role to achieve peace and security, protect democracy, offer grants, feed needy countries in the region and offer technical assistance has not yielded any noteworthy “dividend” to Nigeria or Nigerians in terms of investment opportunities from these benefitting countries nor has it enjoyed local support at home ( Adeniji, 2005; Dan-Fulani, 2014; Folarin, 2013; Jega, 2010). The focusing of Nigeria’s foreign policy on Africa at independence has constituted a huge source of controversy to scholars, analyst and students of international politics. Some reckon that it is a noble course considering the reality and imperatives of the 1960s ( Gambari, 1997; Garba, 1987). Notwithstanding, some scholars submit that it is a diplomatic blunder by a newly independent country whose leaders were unskilled in foreign policy articulation ( Akinboye, 2013; Amao & Uzodike, 2015; Dan-Fulani, 2014; Fayomi *et al.*, 2015; Mailafia, 2010). These scholars, in line with the realist outlook on international politics, believe that nation-building should have been accorded utmost priority through a partnership with developed countries to help the new state realize its full political and economic potential so as to become a haven for the black population all over the world. This is in accord with the realist standpoint which sees interest as the propelling force in international politics conceived in terms of power.

### Nigeria’s foreign policy: A realist world view

Scholars believe that excessive interference in the affairs of other countries and the decision to shoulder the collective burden of the whole of Africa made Nigeria miss opportunities to develop and grow domestically ( Marafa, 2012). This policy thrust has been described as idealistic – hoping for a just, equitable and peaceful world. Thus, over the years, Nigeria ran its foreign policy like an international non-governmental organization (INGO), fostering negro-brotherhood and morality ( Dan-Fulani, 2014). Afrocentrism ultimately drained funds that would have set the new nation on the right footing and propel the new country to greatness. Again, Dan-Fulani (2014) further observed that a realist approach that would guarantee substantial investments in science and technology, thereby moving the economy away from its agrarian nature would have been pursued and efforts made towards national unity. Besides, Nigeria’s alignment to the West, in practice, made it lose opportunities from the East which came with better conditions. 

From this realist viewpoint, it is regrettable that Nigeria has not deployed aid as one of its arsenals in economic diplomacy in its quest for national interest, neither has it deployed it as a tool for imperialism. In her membership and participation in regional organizations, Nigeria willingly shouldered, over and above, what was required of it. These extra burdens can be seen in the country’s statutory contributions to these regional groups, which she accepted, as commensurate with its perceived status. By way of example, Nigeria accounted for between 8 to 10% of the OAU’s regular budget. The same applies to its contribution to the African Development Bank (ADB) where she exerted herself “further by establishing a Trust Fund (NTF) in 1976 with an initial capital outlay of $80million. By December 1990, the NTF had financed 43 development projects in 17 sub-Saharan African states with a total value of $240.764 million” ( Bukarambe, 2000:110). Nonetheless, in 1995, other African states voted against Nigeria’s candidate for the Presidency of the bank, a move that was engineered by the non-regional members of the bank. A source from the National Institute for Policy and Strategic Studies (NIPSS) (Group A) substantiates the above submission when he averred:

*AU is largely funded by 5 countries in Africa out of the 54* [member nations] … *5 countries contribute about 75% of the funding of AU, including Nigeria. But do you know that the other 4 have their citizens in strategic positions in AU except for Nigeria? So, how can you be spending such monumental resources and you cannot push your people further? Nigeria’s interest in this regard needs to be redefined. But then, the redefinition can only come when you have enlightened leadership. That is where the challenge lies* (Respondent A, personal communication, September 21, 2017) *.*

Although the current President of the African Development Bank, Dr Akinwunmi Adesina, is a Nigerian, this is still a drop in the ocean.

On the country’s image problem, Warner (2017:9) contends, “Nigeria’s poor international reputation has led to a distinct dearth of soft power and resultantly, legitimacy problems, which it has assiduously sought to downplay or explain away”. The situation is so appalling that citizens of small neighbouring countries like Chad, Cameroon and Niger attack and harass communities along the borders with a great sense of impunity ( Folarin, 2013). To underscore the importance of this point, Mailafia (2010:182) infers, “it is a paradox that while the country continues to expand its financial support to other countries, its image in the world continues to dwindle.” Nigeria’s image problem has so far defied all remedies as countless man-made and natural disasters continue to plague the country from all corners. Various factors lie at the centre of Nigeria’s lack of legitimacy in the international system. These include bad leadership, economic mismanagement, endemic corruption, ethno-religious violence and general perception, in some quarters, of Nigerians as arrogant, brash and loud ( Adebajo, 2008; Warner, 2017). These, among other factors, have combined to diminish Nigeria’s prestige and weaken its influence in regional and global affairs. Mustapha (2008:52) captures the implication of this image problem concisely, “Nigeria’s national reputation or identity has a bearing on its foreign policy… Simply stated, Nigeria’s national reputation in the international arena as a country of alleged fraudsters and drug barons makes some of its national foreign policy objectives very difficult to attain.” This factor continuously stands in the country’s way in its search for global recognition and power.

Moreover, Akinterinwa (2012) and Folarin (2013) both observe that Nigeria’s first enemies are those countries that have benefitted from her Africa first policy. Akinterinwa (2012) makes particular reference to Nigeria’s bid for the non-permanent seat of the UNSC in 2009 where Liberia, Sierra Leone and Togo, who were not candidates for the position, voted for themselves – a case of discarding their votes instead of casting it for Nigeria, their “Big Brother”. Other cases of ingratitude for Nigeria’s benevolence in her Africa first policy include Ghana (Nigeria supplies electricity on its behalf to Togo and Benin), South Africa (For whom Nigeria made enemies of erstwhile friends as it fought to liberate SA from apartheid), and Egypt (Nigeria mobilized support for it during the 1973 Yom Kippur war). These countries have been contesting for the proposed UNSC permanent seat against Nigeria’s ambition. Folarin (2013) adds that the so-called big powers in international politics today, use such soft economic and socio-cultural diplomacy to accentuate their indisputable hegemony rather than wasting resources on countries that will subsequently turn against them. Another respondent from NIPSS (Group A) makes the following submission on the level of ingratitude Nigeria experiences from its beneficiaries:

*Nigeria has been playing a fatherly role in Africa, but what has Nigeria gained? Nothing! When the goodies are been shared, Nigeria is been relegated, but when the work is needed, Nigeria comes to do the work and at the end of the day what do we have to show for it?* (Respondent C, personal communication, September 17, 2017).

As time went by, Nigeria’s mediatory role on the continent began to reduce and be taken for granted. For example, the 1976 issue between Kenya and Uganda (both members of the East Africa Community, EAC), where Kenya denied the landlocked Uganda access to its ports. Nigeria joined Uganda in pleading to Kenya to restore the latter’s access to the ports all to no avail. However, immediately after Henry Kissinger, America’s Secretary of State stepped into the conflict, Kenya relented and reopened the ports within 48 hours. Commenting on the development, Major General Joseph Nanven Garba, Nigeria’s External Affairs Minister at the time, observed that he was “amazed by the swiftness with which this promise was carried out; within 48 hours, the blockade had been lifted” ( Bukarambe, 2000:111). A similar experience happened when Nigeria tried to mediate in the Ogaden War between Ethiopia and Somalia, and between Zaire and Angola over Shaba. In both cases, the disputing parties appeared to be receptive of Nigeria’s intervention but ended up resolving the issues by taking up arms against each other with the connivance and assistance of foreign powers. These are all clear indications that Nigeria’s perceived “Big Brother” status is hardly recognized and acknowledged by fellow African states. Why you may ask? The accepted linkage between domestic and foreign policy helps in explaining Nigeria’s failure to achieve the long-desired leadership role in Africa.

### Factors responsible for Nigeria’s dwindling regional hegemony

Scholars and respondents have been quite unanimous in adducing reasons for Nigeria’s waning regional influence – leadership failure, corruption, weak economic structures, ethnic and sectarian crisis, infrastructure decay and so on have made Nigeria an object of ridicule in the international circles, eclipsing her erstwhile appreciable image ( Ade-Ibijola, 2015; Folarin, 2013; Jega, 2010; Mbara, 2019; Warner, 2017). These conditions have negatively constrained Nigeria’s foreign policymaking and implementation and reduced her claim of Pax-Nigeriana to an illusory hegemony in the region. Nigeria’s illusory hegemony has elicited a negative response from sister regional members instead of strengthening her claim to the status. Although Nigerian leaders and some Nigerians still believe in Nigeria’s leadership role in Africa, many scholars and observers think otherwise ( Adeniji, 2005; Mbara, 2019; Saliu & Omotola 2008; Warner, 2017). Poverty, insecurity, tribalism and religious bigotry have affected the citizens’ support for their nation’s diplomatic engagements at the international scene and fundamentally constrained her claim to hegemonic status.

The following discussion is instructive given that power theory, which this study is built around, contends that state actors in the international system are principally motivated by national interest, which they feel morally obliged to pursue. In the case of Nigeria, however, the country’s pacifist foreign policy saw her lose the oil-rich Bakassi Peninsula to Cameroon in 2002 through the ruling of the International Court of Justice (ICJ), and Equatorial Guinea took Nigeria to task over territorial waters in the Atlantic among other known affronts against the “Giant of Africa”. Nigeria could have used its diplomatic weight within the continent to deter such insults ( Folarin, 2013). Scholars agree that the fear of Nigeria has long ceased to be the first step to wisdom for many African countries who now take for granted Nigeria’s defeatist, pacifist and feeble approach to dealing with regional issues because of its self-proclaimed commitment to African brotherhood and good neighbourliness ( Amao & Uzodike, 2015; Folarin, 2013; Mbara, 2019). The pertinent question is, where does the interest of Nigerian citizens feature in all of this? Which sane country cedes part of its territory and population to another country, not to mention a viable part for that matter? Was the ceding of Bakassi in the interest of the ordinary citizen or the interest of the political elites? *Vox populi* suggests that President Obasanjo ceded the territory to launder his image in the international community, a case where the interest of a leader becomes the interest of the people. This is the epitome of economic miscalculation and political rascality. Nigeria’s sovereignty has been undermined on all fronts.

Additionally, Mailafia (2010) and Dan-Fulani (2014) submit that national interest which is the guiding principle of the realist foreign policy has no doubt, been absent in Nigeria’s foreign policy endeavours since 1960. Dan-Fulani notes that the country’s membership of many bilateral and multilateral agreements at the sub-regional and regional levels have reduced the country to “a beast of burden” yoked with responsibilities that have no empirical bearing on the country’s national interest in this transient world. Furthermore, Nigeria, he notes is contributing more than its fair share in the sustenance of the African Union’s headquarters in the Ethiopian capital and has played a key part in the Unions specialized agencies and programmes not forgetting the enormous sacrifice it makes in peace operations around the world. If the AU headquarters was situated in Nigeria, perhaps it would have created jobs for the ordinary Nigerians thereby contributing to the economy and giving the country more relevance in global politics. Instead, such opportunities are being sponsored in another country.

### Diplomatic soldering and Nigeria’s strategic interests

Nigeria’s diplomatic activities in the international scene have been bereft of logic and national interest. For instance, Dan-Fulani (2014) describes the practice of maintaining full diplomatic missions in most African countries as a waste of economic and diplomatic resources. The same applies to the country’s membership of numerous international organizations that hardly has any benefit to the people ^2^. Most of the missions on the continent have no strategic importance to the country, but only goes to serve her ego as the “Big Brother”, a status that has is hardly acknowledged by sister nations. To make matters worse, most of these countries do not have more than two dozen of Nigerians doing serious business in them and repatriating profits home neither is the mission serving any strategic interest bordering on our national security. In principle, Nigeria can make do with a maximum of twelve missions in Africa to achieve its goals in the region. Nigeria’s Consulate in South Africa can meaningfully manage the interest of the country in the entire Southern Africa Development Commission (SADC) axis; while the mission in Egypt will manage the country’s concerns in North Africa and parts of the Middle East. In the East African states, Nigerian High Commission in Uganda or Kenya can cater for the country’s interest in the sub-region. The country’s missions in immediate neighbours ^3^ can be maintained for strategic and economic considerations. Around the West African sub-region, Nigeria’s missions in Ghana, Cote d’Ivoire and Senegal can be maintained for shared economic and strategic interests within the sub-region, as well as, geographical proximities ( Dan-Fulani, 2014).

Another diplomatic endeavour that has not yielded commensurate gains for Nigeria is the country’s ECOWAS philosophy. Besides the general protocol regulating relationships, Nigeria is into several bilateral agreements with most countries in West Africa which have been lopsided. Nigeria solely financed the peacekeeping operations in Liberia and Sierra Leone and contributed 95% of the troops that executed the mission. To date, the precise amount spent on the mission has not been published – classified military information perhaps – but estimates show that millions of dollars (taxpayers’ money) were spent for this venture, which had no strategic nor economic interest to Nigeria. Strategically speaking, both Liberia and Sierra Leone have little geographical proximity with Nigeria as there are countries between them and the Atlantic Ocean covering thousands of miles in between. This reduces the probability of high flux of refugees or cross-border militia operations. In the area of economy, Nigeria’s military government at the time had no strategic plan on how to exploit the business opportunities presented by the intervention; there were no plans on how to take over mines and win contracts for the reconstruction efforts after the war. There were no plans for the post-war period which would serve Nigeria’s national interest ( Dan-Fulani, 2014). Besides that, these missions lasted for years without UNSC recognition, financial or logistics support as it is the tradition with United Nations peacekeeping missions. Validating the above argument, another source from NIPSS avers:

*…You go to ECOWAS as a Nigerian, send in an application to go and work there. ECOWAS is 75% funded by Nigeria but the workforce comes from outside Nigeria. But in other climes, countries use their economic power to gain foreign recognition. When the US wanted Boutros-Ghali to leave as UN Secretary-General, it mainly withheld its annual contribution to the UN and the UN almost went bunkers, Boutros had to go. So, you cannot control such monumental economic influence, not to the advantage of your citizens.* (Respondent D, personal communication, September 24, 2017)

In recent years, Ghana has exhibited hostile attitudes towards Nigeria and Nigerians living in the Republic. Policies to beat back Nigerian traders led the to the legislation that foreigners who wished to do business in the country must show a proof of 300,000 USD in its company account. This violates the ECOWAS protocol on free trade and market integration. These xenophobic attitude towards Nigeria and Nigerians was heightened on the night of 19 June 2020, when Nigeria’s High Commission in Ghana was demolished “under the full protection of State Security,” an act of naked aggression against the Nigerian state and in violation of Article 22 of the Vienna Convention on Diplomatic Relations ( “Demolition of Nigerian High Commission,” 2020). Correspondingly, Nigeria has been supplying Niger Republic electricity (an essential commodity at home) since 1960 at a very subsidized rate. This is sequel to an agreement reached by the two countries in the 1960s, which prevented Niger from building a dam on the River Niger as it will obstruct the flow of water to the Kainji dam. Six decades later, it is time to review the agreement to reflect the current global economic realities.

Against these backgrounds, there is a need to ask some critical questions: how has Nigeria’s foreign policy benefited the ordinary citizens of the country? Has the welfare and bare necessities of life of the common person been captured thus far? Most respondents strongly believe that foreign policy formulation and execution in Nigeria is an elitist affair. A respondent from group B maintains: *When you talk of foreign policy, it has always been propagated by the elites.* (Respondent B, personal communication, September 7, 2017). In the same way, respondent E maintains:

*Nigeria’s foreign policy is more like deceit to the people in the sense that what Nigeria presents as foreign policy has never reflected on the people… See the case of Nigeria in Liberia and Sierra Leone in the late 1980s, [ECOMOG] See the way Nigerians are treated by these countries today… Let me be frank with you, it is total deceit [foreign policy]. Nigeria is a property owned by a few individuals* (Personal communication, September 15, 2017).

### Elitist considerations in Nigeria’s foreign policy

In Nigeria (and most African countries), politics has become fierce and bloody, as it grants the winner control over state resources. This partly explains why those occupying state political positions cling to power so tenaciously that they even deny the losers their unalienable right. Under such circumstances, development as a process is absent from the manifesto of those in authority. Rather, their parochial interests are seen as development priorities of the people and foisted on them ( Nuamah, 2003; Omoweh, 2000).

The elites have hijacked the state apparatus in Nigeria, thereby retarding the country’s quest for Pax-Nigeriana ( Adebajo, 2008; Nuamah, 2003; Wright & Okolo, 1999). Analysing Nigeria’s foreign policy and domestic conditions, misdemeanours, Adebajo (2008:24) in his classic work, “Hegemony on a shoestring: Nigeria’s post-Cold War foreign policy” in *Gulliver’s Troubles*, compares Nigeria to a potentially wealthy Gulliver, and its leaders as the Lilliputians, “whose petty ambitions and often inhumane greed… have prevented a country of enormous potential from fulfilling its leadership aspirations and developmental potential.” Nigeria’s foreign policy averred Akinterinwa (2004) has never reflected the needs of the ordinary citizens; rather elitist considerations inspire its formulation, articulation and implementation. Thus, the needs of the cream of the society – the business class, bureaucrats, military and civil rulers are reflected in the country’s foreign policy. Corroborating this view, Adeniji (2004) submits that the common citizen in Nigeria has not been the focus of policy. The law, he notes, has been the focus, not the people. The people who made the law must always be placed above it in order of importance and defending a state whose citizens are worthless is equally useless. Likewise, Alh. Sule Lamido, Nigeria’s former Minister of Foreign Affairs observed that there is a disconnect between the public and foreign policy formulation process in Nigeria as “the people on the street” are left out in the decision-making process. Consequently, the Nigerian public has developed “policy apathy” towards government policies. It was for this reason, he notes, that the debt relief granted Nigeria in 2005 was not celebrated by the people on the street and many exhibit hostile tendencies towards Nigeria’s bid for a permanent seat on an expanded UN Security Council ( Uhomoibhi, 2012). Corroborating this view, Warner (2017:10) asserts, “Nigerian civil society’s indifference toward the pursuit of Pax-Nigeriana could be at the heart of the country’s inability to achieve it.”

Irrespective of the flaws in Nigeria’s foreign policy over the years, in fairness, this study also discovered that the outcome has not been a total failure as some respondents maintain. Citing Nigeria’s intervention in various crisis in West Africa (Liberia, Sierra Leone, Cote d’Ivoire, Mali and Gambia), respondent F from NIPSS submits that the benefits of these interventions:

… *Might not be clear at the immediate, but there is certainly something Nigeria has benefitted. By ensuring stability in the West African sub-region, Nigeria has also remained stable. When Liberia collapsed, more than 45% of the refugees came into Nigeria. However you look at it, it* [the influx of refugees] *had a negative impact on the Nigerian economy. In the mid-1970s and early 80s, when the Ghanaian economy crumbled, what was the next destination – Nigeria! … Virtually all Nigerian universities were taken over by Ghanaian scholars, some of them still here. Go to the University of Calabar, OAU [Obafemi Awolowo University] … Even if there appear to be no immediate gains for Nigeria when we look at it strategically. Nigeria has also succeeded in saving itself from the unnecessary influx of refugees. Look at the Great Lakes region in the Horn of Africa – DRC, Burundi, Rwanda, to some extent Kenya and Uganda. They have all been involved in one complex crisis threatening human existence. The country that has suffered it is Tanzania because of its stability. Tanzania has always at every point in time taken the chunk of the refugees that leave these countries. This has impacted significantly on the Tanzanian economy [See also the case of Zimbabwe and South Africa]. So, these are some of the things Nigeria may have benefitted [from its Africa first foreign policy]. But besides it, there is a need for Nigeria to redefine what its strategic interests are, especially as it has to do with its citizens in all its foreign engagements. It has to be clearly defined. Nigeria cannot be losing human and material resources of monumental magnitude only for its citizens to be treated with disdain in these countries. You cannot do that to the USA. If we are not seeking for spheres of interest or territories to occupy, we can at the international level gain some regard and respect for our citizens* (Personal communication, September 18, 2017).

The Afrocentric policy without Nigerians is sterile. No matter how successful foreign policy is, without Nigerians as the immediate beneficiaries, it will likely not win the people’s support. To fill the gap, Uhomoibhi (2012) postulates a constructive and beneficial concentricism.

### Transition from economic diplomacy to citizen-centred diplomacy

It was perhaps in response to these crucial questions and the image crisis the country was grappling with that Prof Dora Akunyili, Nigeria’s former Minister of Information and Culture, launched Nigeria’s rebranding campaign. Along with this came the need to reposition the country’s foreign policy. These two areas were given priority by the Umaru Musa Yar’Adua’s administration who believed that addressing the socio-economic and political problems in the country was a critical necessity. Moreover, Dan-Fulani (2014) observes that three major events in the international scene were also responsible for this move: the end of the Cold War, successful decolonization of Africa and the end of apartheid in South Africa made Afrocentrism an anachronistic idea in Nigeria’s foreign policy. The variables that necessitated this policy stand have given way to new challenges that require a complete repositioning of Nigeria’s foreign policy objective.

Late Chief Ojo Maduekwe, Nigeria’s former Minister of Foreign Affairs during the Yar’Adua’s government, was credited for introducing this citizen diplomacy. Justifying its introduction, Maduekwe observed that as the largest concentration of Black people on earth, it behoved on the country to stand as a symbol of the black success story. Hence, citizen diplomacy suggests using foreign policy as a powerful weapon to showcase to the world who Nigeria and Nigerians are. It also implies that the global community must assume responsibility for its actions towards Nigerians in all circumstances ( Maduekwe, 2007). Lending credence to the new diplomacy, Folarin (2013) supports this new policy position and Nigeria’s demand for respect from the international community by noting that the country’s large population, economic hegemony on the African continent and diplomatic track-record continentally and globally are enough credentials for it to be accorded the respect it demands and deserves.

Conversely, as laudable as this new citizen diplomacy may sound, scholars have been quite sceptical of its execution. Dickson (2010) cited in Amao & Uzodike (2015) observed that in 2007, a career diplomat from Nigeria, Dr Ngozi Ugo was nominated for the position of the Secretary General’s Deputy Special Representative and an Ombudsman at the UN. For her nomination to be confirmed, she required the diplomatic endorsement of her country’s government. However, owing to official bureaucracy and sheer incompetence from the Ministry of Foreign Affairs, she lost the position as time lapsed. What manner of citizen diplomacy is Nigeria practising when it cannot protect the interest of its citizens? Her presence would have boosted Nigeria’s quest for a UN permanent seat on a reformed Security Council and other agencies of the UN. Further, Dickson notes:

Other more serious countries campaign for their citizens and that is why the highest-ranking African in the UN system is a Tanzanian woman. Go to the Commonwealth Secretariat in London you may think you are in India’s Ministry of Foreign Affairs because of the number of Indians there. And this is where our own Chief Anyaoku served for almost four decades. When is Nigeria going to stand and recognize its own? It is sad, unfortunate and indeed painful (cited in Amao & Uzodike, 2015:12).

Additionally, a series of examples abound where the Nigerian government has failed its citizens within the era of the new policy dispensation. Among them is the ceding of the oil rich Bakassi local government to Cameroon following the ruling of the ICJ. While the transfer of the territory is still in process, there has been massive reports of abuse, molestations and harassments from the Cameroonian authorities against Nigerians living in the area which is their ancestral home ( Folarin, 2013:9). Others are the series of xenophobic attacks on Nigerians living in South Africa in 2008, 2015, 2017 and 2019.

While scholars and foreign policy analysts believe that citizen diplomacy represents a radical shift away from the Africa-centred thrust, others suggest that the government may have only succeeded in achieving policy documentation rather than the actual execution. There is a need, therefore, for the government to be more proactive and responsive, both in words and deeds, to the predicaments of its citizens as the nation gravitates to the “people-first approach” diplomacy. Nigerians everywhere must-see sincerity in their government to protect and enhance their welfare.

## Conclusion

This investigation made a retrospective look at Nigeria’s foreign policy in the last 55 years. The focus has remained constant – Africa and the senseless generosity to sister countries. Most supervising heads coin concepts that have no meaning or relevance in international relations and foreign policy articulation. As Nigeria pursued a busybody foreign policy, other countries with similar power base refused the temptation of extraterritorial activism within the region or elsewhere and chose to consolidate their power base for the greater good of their people. At independence, countries like Cote d’Ivoire and Senegal had a reasonably stable economy but chose to pursue foreign policies that would benefit their people through the Non-Aligned Movement (NAM). This study aligns with that of Jega (2010:7) who notes that despite significant external and domestic constraints, one sees much to be proud of as there are “notable and noteworthy accomplishments if only lessons could be learnt and reform initiatives launched appropriately.”

## Data availability

### Underlying data

Access to the interviews is restricted since they identify each respondent. However, researchers who wish to perform further analysis can make an application to the Humanities & Social Sciences Ethics Committee, University of KwaZulu-Natal, Westville Campus, Govan Mbeki Building, Private Bag X54001, Durban, 4001, South Africa for access to the data. Email: ximbap@ukzn.ac.za.

### Extended data

Figshare: INTERVIEW SCHEDULES. https://doi.org/10.6084/m9.figshare.12609353.v2 ( Mbara, 2020).

This project contains the questions asked to each group of participants during the key informant interviews.

Data are available under the terms of the Creative Commons Attribution 4.0 International license (CC-BY 4.0).
