# Supplementary material for: Afrocentrism, national interest and citizen welfare in Nigeria’s foreign policy maneuvers
Source: F1000Res. 2020 Aug 18;9:997. [Version 1] doi: 10.12688/f1000research.25036.1 (PMC8256188; doi:10.12688/f1000research.25036.1)
Supplement: Peer review history

# Peer review: Reviewer response for version 1

**DOI:** 10.5256/f1000research.27619.r86788

**Published:** 2021-07-02

**Authors:** Stephen Osaherumwen Idahosa

**Affiliations:** 1 Department of Theory and History of International Relations, Peoples''s Friendship University of Russia, Moscow, Russian Federation

I wish to thank the editors of the “ *F1000Research*” for the opportunity they accorded me, to read the article “Afrocentrism, national interest and citizen welfare in Nigeria’s foreign policy maneuvers”. I found this work an interesting paper which deals with a contemporary and important debate surrounding Afrocentrism - Africa as the centrepiece of Nigeria’s foreign policy. In particular, by focusing its attention on Nigeria’s generosity and developed an analysis through the lens of the realist theoretical framework - the author states that “Regardless of these successes, this Africa-centred foreign policy concentration has not been without flaws. From this realist viewpoint, it is regrettable that Nigeria has not deployed aid as one of its arsenals in economic diplomacy in its quest for national interest, neither has it deployed it as a tool for imperialism” and that “Nigeria’s bid for the non-permanent seat of the UNSC in 2009 where Liberia, Sierra Leone, and Togo, who were not candidates for the position, voted for themselves – a case of discarding their votes instead of casting it for Nigeria, their ‘Big Brother’. Other cases of ingratitude for Nigeria’s benevolence in her Africa first policy include Ghana (Nigeria supplies electricity on its behalf to Togo and Benin), South Africa (For whom Nigeria made enemies of erstwhile friends as it fought to liberate SA from apartheid), and Egypt (Nigeria mobilized support for it during the 1973 Yom Kippur war). These countries have been contesting for the proposed UNSC permanent seat against Nigeria’s ambition.” In the end “Afrocentrism ultimately drained funds that would have set the new nation on the right footing and propel the new country to greatness.” This is an interesting claim that pushes me to support this article.

 A few suggestions:

- The work is clearly and accurately presented, however, concerning the literature, I think there are enormous books written by retired Ambassadors that could be useful.
- The study design is appropriate and it does have academic merit and the data could be replicated. In a further study, I would suggest that the conclusion be expanded to reflect more on the result and possible recommendations.
- The research possesses more strengths than weaknesses. However,  areas such as the contemporary state of funding of the Foreign Service and the lack of appropriate capital for the vehicular instrument - MFA, to ensure the implementation of Nigeria's Foreign policy is either scantly written on or never written on at all. I think these areas should be explored as they play a huge and enormous role in the success and discharge of Nigeria's Foreign Policy.

Therefore, suffice it to state that, the article has much potential to be indexed as it pays attention to an area that is significantly important in Nigeria’s foreign policy, its articulation, and implementation. The paper is well written, well organised, and developed. I recommend it for indexing in this current form. Congratulations.

Is the work clearly and accurately presented and does it cite the current literature?

Yes

If applicable, is the statistical analysis and its interpretation appropriate?

Yes

Are all the source data underlying the results available to ensure full reproducibility?

Yes

Is the study design appropriate and is the work technically sound?

Yes

Are the conclusions drawn adequately supported by the results?

Yes

Are sufficient details of methods and analysis provided to allow replication by others?

Yes

Reviewer Expertise:

Regional security, insurgency, spread of instability in the Sahel and Lake Chad regions, insecurity, terrorism and foreign policy.

I confirm that I have read this submission and believe that I have an appropriate level of expertise to confirm that it is of an acceptable scientific standard.

# Peer review: Reviewer response for version 1

**DOI:** 10.5256/f1000research.27619.r84323

**Published:** 2021-06-10

**Authors:** Ismail Bello

**Affiliations:** 1 Department of International Relations and Diplomacy, Baze University, Abuja, Nigeria; 2 Department of Political and Adminstrative Studies, Kampala International University, Kampala, Uganda

This paper contributes to the discussion on Nigeria's Afrocentric foreign policy which has been in place since 1960. The paper further examines the impact of this policy on the welfare of the common man in Nigeria. As stated in various foreign policy textbooks, foreign policy of each states should be premised on National Interest, which is basically the interest of the state and its citizens. This paper submits that the afrocentric policy played a key role in liberation and anti-aparthied struggle in Southern Africa. Howerever, policy leaders have often neglect their first responsibility which is to provide for Nigerians and improve on citizens welfare. This perhaps influenced  the adoption of citizen diplomacy by late president Umar Musa Yaradua. The paper concludes that Afrocentric foreign policy adopted by the Nigerian is more of senseless generosity. This conclusion is in line with my thoughts that the Afrocentric policy is a Father Christmas diplomacy which hasnt yielded much to the Nigerian state.

Is the work clearly and accurately presented and does it cite the current literature?

Yes

If applicable, is the statistical analysis and its interpretation appropriate?

Not applicable

Are all the source data underlying the results available to ensure full reproducibility?

Yes

Is the study design appropriate and is the work technically sound?

Yes

Are the conclusions drawn adequately supported by the results?

Yes

Are sufficient details of methods and analysis provided to allow replication by others?

Yes

Reviewer Expertise:

Nigerian Foreign Policy, Multinational Corporations, and Sustainable Development Goals.

I confirm that I have read this submission and believe that I have an appropriate level of expertise to confirm that it is of an acceptable scientific standard.
